# The usefulness of modified national early warning score with the age level in critically ill medical patients

**DOI:** 10.1186/2197-425X-3-S1-A834

**Published:** 2015-10-01

**Authors:** YS Lee, JW Lee, J Lee, NE Min, JE Park, JW Jung, DI Park, KD Kim, HJ Ahn, JW Choi, Y-H Park, S Ryu, WJ Jeong, JY Moon

**Affiliations:** Division of Pulmonology, Department of Internal Medicine, Severance Hospital, Yonsei University College of Medicine, Seoul, Korea, Republic of Korea; Daejeon Regional Emergency Center, Chungnam National University Hospital, Daejeon, Korea, Republic of Korea; Division of Intensive Care Units, Department of Nursing Care, Chungnam National University Hospital, Daejeon, Korea, Republic of Korea; Division of Pulmonary and Critical Care Medicine, Department of Internal Medicine, Chungnam National University Hospital, Daejeon, Korea, Republic of Korea; Department of Emergency Medicine, Daejeon Sun-Hospital, Daejeon, Korea, Republic of Korea; Department of Emergency Medicine, Chungnam National University Hospital, Daejeon, Korea, Republic of Korea

## Introduction

Early warning scores are recommended as a part of the early identification and intervention to patient deterioration. The National Early Warning Score (NEWS) allows early recognition of patient deterioration, and has the role of prognostic predictor. In addition, age is the most important factor to influence the mortality and prognosis in inpatients.

## Objectives

To investigate whether the predictive value of the NEWS could be improved by including the factor of age, and to compare the modified NEWS with the pre-existing NEWS system.

## Methods

This is a retrospective study on 1558 patients, who were screened by NEWS during admission period, between December, 2013 and March, 2014. The score of modified NEWS was NEWS plus the score of age level. The score of age level was defined as follows: 10-19 years, 1 point, 20-29 years, 2 points,; 30-39 years, 3 points; 40-49 years, 4 points; 50-59 years, 5 points; 60-69 years, 6 points; 70-79 years, 7 points; 80-89 years, 8 points; 90-99 years, 9 points. We analysed hospital mortality, 30 day mortality, 90 day mortality, and intensive care unit (ICU) admission as endopoints for identifying the predictive value of the modified NEWS. The correlation between APACHE II score and modified NEWS in patients, who were transferred to the ICU, was also analysed. Logistic regression analysis was performed to identify important factors for predicting hospital mortality.

## Results

The median age was 63 years old and 57.2% of the patients were male. The median score of modified NEWS was six and 4.8% of the patients were transferred to the ICU during screening. The modified NEWS was better than NEWS to predict hospital mortality, 30 day mortality, 90 day mortality, and ICU admission (area under the receiver operating characteristic curve, 0.808 vs. 0.795 in hospital mortality; 0.710 vs. 0.643 in 30 day mortality; 0.686 vs. 0.655 in 90 day mortality; 0.774 vs. 0.765 in unanticipated ICU admission). In multivariate logistic regression, the odds ratio of modified NEWS were 1.3 (95% confidence interval 1.16-1.39. p < 0.001) in all individuals with hospital mortality. In addition, modified NEWS correlated significantly with APACHE II score (r = 0.420, p < 0.001) in patients, who were transferred to the ICU.

## Conclusions

The predictive power of the modified NEWS for prognosis was better than that of NEWS. More studies on larger numbers of the patients are warranted.
